# Evaluating the Diagnostic Potential of Four-Dimensional Flow Magnetic Resonance Imaging in Aortic Stenosis Diagnosis: A Systematic Review and Meta-Analysis

**DOI:** 10.7759/cureus.73339

**Published:** 2024-11-09

**Authors:** Santosh K Chandrasekar, Mahesh Kolli, Agnes George, Dhanush Kodali, Harisha Nagaraja Shivamoggi, Shyam Nikethen Girivasan

**Affiliations:** 1 Department of Medicine, University Hospital Ayr, Ayr, GBR; 2 Department of Telemedicine, Apollo Hospitals, Chennai, IND; 3 Department of Neurology, Baby Memorial Hospital, Calicut, IND; 4 Department of Pharmacy, Jagadguru Sri Shivarathri (JSS) Academy of Higher Education &amp; Research, Ooty, IND

**Keywords:** 2d mri, 4-dimensional flow mri, aortic stenosis (as), transthoracic echocardiography (tte), valvular heart disease

## Abstract

This systematic review and meta-analysis evaluates the potential of four-dimensional flow magnetic resonance imaging (4DFM) in assessing aortic stenosis (AS) compared to traditional imaging modalities like two-dimensional phase contrast MRI (2D MRI) and transthoracic echocardiography (TTE). AS is a common and severe valvular heart disease, particularly in older adults, requiring accurate diagnosis for proper clinical management. Conventional imaging methods have limitations in capturing complex flow dynamics, prompting the need for advanced modalities like 4DFM. The objectives of the review were to determine whether 4DFM offers superior diagnostic metrics, including peak aortic jet velocity, transvalvular pressure gradients, and aortic valve area (AVA), and to identify potential advantages of 4DFM in overcoming the limitations of traditional modalities. This review included six cohort studies with 285 participants, examining the diagnostic accuracy of 4DFM in terms of peak aortic jet velocity, transvalvular pressure gradients, and aortic valve area (AVA). Studies were selected from MEDLINE (PubMed), Cochrane Library, and Google Scholar databases between December 2010 and October 2024. The study pool was limited by stringent inclusion criteria focusing on cohort studies that directly compared 4DFM with TTE or 2D MRI for AS assessment. The National Institutes of Health Quality Assessment Tool and Cochrane ROBINS-I tool were used to assess bias. Quantitative results showed that 4DFM typically measured higher AVA values than TTE, with a mean difference of 0.48 cm² (95% CI: -0.16 to 1.12). For mean pressure gradients, 4DFM reported slightly higher measurements in individual studies, but pooled results showed no significant difference compared to TTE (mean difference: 3.32 mmHg, 95% CI: -2.30 to 8.93). In terms of peak aortic jet velocity, 4DFM demonstrated a pooled mean difference of -0.18 m/s (95% CI: -0.44 to 0.08) compared to TTE. High heterogeneity was observed across studies (e.g., I² = 97% for peak velocity, I² = 93% for AVA), likely due to differences in patient populations, imaging protocols, and software for data analysis. 4DFM demonstrates potential as a complementary imaging tool, particularly in complex AS cases where conventional methods like TTE may provide inconclusive results. Its capacity to capture intricate flow dynamics and deliver high spatial resolution could inform clinical decision-making, potentially influencing practice guidelines to integrate 4DFM as a supplementary tool. Limitations such as high costs, specialized training requirements, and access challenges currently restrict widespread adoption. Limitations of this review include small sample sizes, high heterogeneity, and variability in patient populations and imaging protocols. Despite these challenges, 4DFMI demonstrated superior spatial resolution and complex cardiovascular flow assessment, suggesting it could serve as a valuable complement to TTE for more detailed AS evaluation, particularly in complex cases. Future studies should aim to standardize imaging protocols, incorporate larger and more diverse populations, and conduct cost-benefit analyses to support the integration of 4DFM into clinical practice, potentially shaping future diagnostic guidelines.

## Introduction and background

Magnetic resonance imaging (MRI) has been a major advancement in the clinical estimation of diseases associated with heart and blood vessels since the late 1980s, and quantitative flow imaging is now used routinely throughout cardiothoracic and vascular assessments [1]. A major improvement in the four-dimensional flow MRI (4DFM) is encoding three-directional velocities in three spatial dimensions during the cardiac cycle [2]. As a non-invasive procedure, it helps in offering fundamental information on blood flow in different areas especially in cardiothoracic and cerebrovascular diseases [3]. Derived from phase contrast MRI, 4DFM provides good vascular depiction with flow quantification to assess cardiovascular flow dynamics through the acquisition of time-varying three-dimensional flow of blood in the heart and great vessels [4].

Transthoracic echocardiography (TTE) is an imaging study of the heart without the insertion of catheters or other instruments into the body [5]. TTE is one of the initial imaging modalities because it facilitates real-time visualization of cardiac chambers, wall motion, and valve functions and is a key tool in diagnosing heart diseases [6]. TTE's advantages include wide availability, portability, safety, and cost-effectiveness, as it avoids ionizing radiation [6]. TTE has grown over time, with a focus on motion-based echocardiography, which is M-mode that was pioneered by Inge Edler in 1953 [7]. During the 1970s, major improvements in two-dimensional (2D) ultrasound and Doppler opened new ways to observe blood flow and measure the pressure differences in the cardiovascular system, which improved diagnostic medicine [7].

When patients present with new-onset dyspnea, chest pain, or new heart murmurs, TTE is used to screen for aortic stenosis (AS), with recommendations regarding its valvular function and structure [8]. Consequently, TTE has a very crucial role in the clinical assessment of AS [9]. AS is a prevalent valvular heart disease, particularly affecting geriatric populations [10]. It develops from an initial stenosis and mild calcification of the aortic valve to severe calcification of the valve, thus entirely blocking blood flow between the left ventricle and the aorta [11]. The diagnosis of severe AS by Doppler echocardiography is indicated by a mean transvalvular pressure gradient ≥40 mmHg, a peak aortic jet velocity ≥4 m/s, or an aortic valve area (AVA) ≤1.0 cm² [11,12].

Similar to TTE and 4DFM, phase-contrast MRI in two dimensions (2D MRI) is another widely utilized technique in cardiovascular imaging for assessing blood flow velocity and dynamics [13]. 4DFM differs from 2D MRI by capturing time-resolved, three-dimensional blood flow, providing comprehensive visualization and enhanced diagnostic accuracy [13]. Recent studies comparing the efficacy of 4DFM with conventional TTE have highlighted significant findings, where 4DFM consistently yielded higher AVA values compared to conventional methods [14-20]. Accurate assessment of AS severity is vital for timely treatment decisions, potentially improving outcomes through appropriate medical or surgical intervention. While TTE is the cornerstone of AS diagnosis due to its accessibility and cost-effectiveness, integrating 4DFM as a complementary tool provides additional data for nuanced clinical decisions, ensuring a more tailored approach for patients with atypical or inconclusive TTE results. 4DFM, although it offers excellent spatial resolution and can also measure highly complex flow patterns, has not yet gained significant favor in routine clinical applications. This study aims to systematically compare the diagnostic accuracy of 4DFM with its conventional modalities, such as 2D MRI and TTE, in terms of its ability to diagnose accurate AS severity and their ability to provide predictive clinical endpoints and impact the course of future guidelines in clinical treatment of AS.

## Review

Methods

Selection Criteria

We followed the Preferred Reporting Items for Systematic Reviews and Meta-Analysis (PRISMA) guidelines [21] and the Cochrane Handbook [22] for conducting this study and meta-analysis. The Participants, Intervention, Comparisons, Outcomes, and Study Design (PICOS) format was followed as mentioned in the inclusion and exclusion criteria.

Inclusion criteria: The study included participants (P) as patients diagnosed with aortic stenosis (AS) who underwent intervention (I) involving 4D flow MRI (4DFM) as the primary intervention. For comparison (C), traditional imaging techniques such as 2D and 3D Doppler transthoracic echocardiography (TTE) and 2D flow MRI were used. Outcomes (O) assessed included peak aortic jet velocity, mean transvalvular pressure gradient, and aortic valve area. The study design (S) focused on non-randomized cohort studies published between 2010 and 2024. 

Exclusion criteria: Exclusion criteria involved non-human participants and patients without confirmed AS. Studies that utilized diagnostic methods other than 4DFM and TTE were excluded to ensure consistency in evaluating imaging techniques. The exclusion of these methods helped maintain a focus on the key outcomes of interest related to AS. This approach allowed for a direct comparison of 4DFM with widely used diagnostic methods to assess its effectiveness in the clinical evaluation of AS.

AS was selected as the primary focus of this meta-analysis due to its high prevalence and clinical impact, particularly in geriatric population, where accurate diagnosis and assessment are critical for patient management. However, the initial search strategy included broader terms for other valvular conditions, such as mitral or tricuspid valve issues, which may share overlapping diagnostic techniques. To maintain the specificity of our findings, studies involving mixed valvular conditions, particularly those with combined AS and significant regurgitation, were excluded in the final selection to ensure that the analysis reflected the diagnostic efficacy of 4D flow MRI and echocardiography specifically for AS. This approach allowed for comprehensive initial search results while refining the focus on AS in line with the study's objectives. As mentioned in the introduction, a peak aortic jet velocity ≥4 m/s, mean transvalvular pressure gradient ≥40 mm Hg, and AVA ≤1.0 cm² were considered as the acceptable diagnostic ranges used to measure severe AS.

Search Strategy and Study Selection

Two reviewers comprehensively searched for eligible studies from MEDLINE (PubMed), Cochrane Library, and Google Scholar from December 2010 til October 2024, focusing on cohort studies comparing 4DFM with TTE and 2D MRI. No language restriction was applied. Reference lists of all eligible trials were also searched to identify other studies. Duplicate studies were eliminated using EndNote 20.2.1 (Clarivate Analytics, Philadelphia, PA). A description of the keywords with Boolean operators used for each database is provided in Table 1. The chosen databases - MEDLINE (PubMed), Cochrane Library, and Google Scholar - were selected for their extensive coverage of clinical and systematic reviews, providing relevant peer-reviewed studies on imaging techniques. The starting timeframe of December 2010 was chosen to reflect advancements in 4D flow MRI technology post-2010, enhancing the quality and relevance of included studies.

**Table 1:** Search strategy and keywords utilized

Database	Search strategy
PubMed (9 results)	("4DFM"[MeSH] OR "four-dimensional flow MRI" OR "4D flow" OR "4D MRI" OR "4D cardiovascular MRI") AND ("echocardiography"[MeSH] OR "echocardiography" OR "2D echo" OR "3D echo" OR "Doppler echocardiography") AND ("valvular heart disease"[MeSH] OR "heart valve disease" OR "valvular dysfunction" OR "aortic stenosis" OR "mitral regurgitation") AND ("diagnostic accuracy" OR "sensitivity and specificity" OR "predictive value of tests" OR "clinical outcomes" OR "long-term outcomes" OR "valve surgery" OR "survival")
Google Scholar (384 results)	4DFM vs echocardiography "cohort studies"
Cochrane Library (0 results)	("4DFM" OR "four-dimensional flow MRI" OR "4D cardiovascular MRI") AND ("echocardiography" OR "2D echo" OR "3D echo" OR "Doppler echocardiography") AND ("valvular heart disease" OR "aortic stenosis") AND ("diagnostic accuracy" OR "clinical utility" OR "outcomes" OR "valve surgery" OR "long-term survival")

Data Extraction

Two reviewers independently assessed the papers on Mendeley based on their titles and abstracts in accordance with the qualifying requirements. Mendeley was chosen for title and abstract screening due to its efficient reference management capabilities and automatic duplicate detection, which streamlined the initial study selection process. The full texts of the remaining papers were separately reviewed, and disagreements were resolved by a third, blinded reviewer who independently made a final decision on disputed studies, ensuring an unbiased resolution process. Common disagreements arose regarding the inclusion of studies that did not explicitly report AS severity. The following information was retrieved from the studies using a piloted Excel (Microsoft, Redmond, Washington) sheet: the first author's surname, year of publication, country, research type, number of participants (n), mean age, and functional outcomes. Microsoft Excel was used for data extraction because of its flexibility in organizing multiple categories and filtering data, allowing for detailed categorization across study characteristics and outcomes. The data extraction sheet in Excel was pre-tested with a sample of studies to ensure consistency in data entry and category definitions. The sheet included key categories such as author, publication year, study design, sample size, mean age, diagnostic outcomes, and quality assessment scores. Additionally, specific columns were dedicated to quality notes, enabling systematic documentation of methodological rigor and potential sources of bias. The baseline characteristics are described in Table 2, which offers an overview of the major components of the studies included in the meta-analysis, concentrating on the year of publication, research design, sample size, mean age, clinical presentation and therapy, and comparator utilized.

**Table 2:** Characteristics of the studies included AS - aortic stenosis; MRI - magnetic resonance imaging; TTE - transthoracic echocardiography

Study	Year of publication	Study design	Sample size	Mean age	Clinical presentation	Treatment and comparator assessed
Allen et al. [14]	2015	Prospective cohort	30	13.9	pediatric healthy volunteers	4DFM vs ultrasound TTE
Adriaans et al. [20]	2019	Prospective cohort	20	69.3	AS	4DFM vs ultrasound TTE
Archer et al. [15]	2020	Prospective cohort	18	74.2	AS	4DFM vs Doppler TTE
Hälvä et al. [19]	2021	Prospective cohort	90	73.3	90 AS + 10 healthy volunteers	4DFM vs TTE
Huh et al. [18]	2022	Prospective cohort	44	51.9	37 healthy volunteers + 7 AS patients	4DFM vs Doppler TTE
Hautanen et al. [17]	2023	Prospective cohort	83	53.7	AS	4DFM vs 2D phase contrast MRI

Methodological Quality Appraisal

National Institutes of Health Quality Assessment Tool for Observational Cohort and Cross-Sectional Studies [23] consisting of 14 questions was used, each scored as "Yes", "No", or "Not Applicable". The study quality was scored on a scale from zero to 14 and classified as poor, fair, or good based on the total score (zero-seven points as poor, eight - 10 points as fair, or 11-14 points as good). For the NIH Quality Assessment Tool, studies with mixed ratings across domains were assessed holistically. When a study had both "Yes" and "No" responses that created ambiguity in overall quality, an average rating approach was used. "Not Applicable" responses were excluded from the total score calculation to avoid penalizing studies for criteria irrelevant to their design, ensuring fairness in quality assessment. The NIH Quality Assessment Tool was selected for evaluating observational cohort studies due to its emphasis on assessing validity within cohort designs, focusing on factors such as population definition, exposure measurement, and outcome assessment. This tool's criteria align well with the study's goal of ensuring methodological rigor in observational data on imaging modalities. For the included observational or cohort studies, the Cochrane Risk Of Bias In Non-randomized Studies - of Interventions (ROBINS-I) tool [24] was used where the studies were assessed based on the seven domains confounding selection of participants classification of interventions. Deviations from intended interventions, missing data, measurement of outcomes, and selection of reported results. ROBINS-I tool was used alongside the NIH tool to provide a more comprehensive bias assessment, particularly addressing confounding, participant selection, and outcome measurement, which are critical in observational studies lacking randomization. The results were represented through traffic light plots and summary bar charts using the Cochrane Risk-of-bias VISualization (Robvis) tool [25]. In the traffic light plot, green, yellow, and red indicators represented low, moderate, and high risk of bias, respectively, across domains. Expected findings included a low to moderate risk of bias in most domains, with occasional high bias in participant selection. Bar charts summarized these findings, highlighting areas where specific biases were prevalent across studies. To mitigate potential biases, we employed rigorous inclusion criteria, ensured consistency in data extraction, and performed sensitivity analyses wherever feasible.

Outcomes

The results of the studies focused on the mean peak flow velocity, mean pressure gradient, aortic valve area, and intraclass correlation (ICC) coefficient of the radiological findings between 4DFM and the comparator group, such as TTE and 2D flow MRI. Reporting ICC values is essential to confirm the consistency between 4DFM and traditional imaging methods like echocardiography, ensuring clinical reliability in diagnosing AS. 

Statistical Analysis

The data were analyzed using Review Manager version 8.7.0, the Cochrane collaboration (October 3, 2024) [26]. The random effects model was used to calculate mean differences (MD) and their corresponding 95% confidence intervals (CI) except for the parameter mean pressure gradient. The random-effects model was utilized due to the high estimated heterogeneity(>75%) of the true effect sizes. For each synthesis, the I^2^ index and the Chi-squared test (Chi²) were used for the assessment of heterogeneity, and a p*-*value of less than 0.05 was considered critical for the heterogeneity of the included studies. The inverse variance (IV) method was used to combine individual study results. The inverse variance method was chosen for this meta-analysis to account for differences in study sizes and ensure that larger studies with more precise estimates contribute more heavily to the pooled results. This approach assigns greater weight to studies with lower variance, improving the overall accuracy and stability of the meta-analysis findings. A Z-test for the overall effect was conducted, and a p-value of less than 0.05 was deemed statistically significant, demonstrating a difference in the risk between experimental and control groups. The risk ratios were visualized using a forest plot, where an RR of less than 1 indicated a reduced risk in the experimental group. 

Results

Study Characteristics

**Figure 1:**
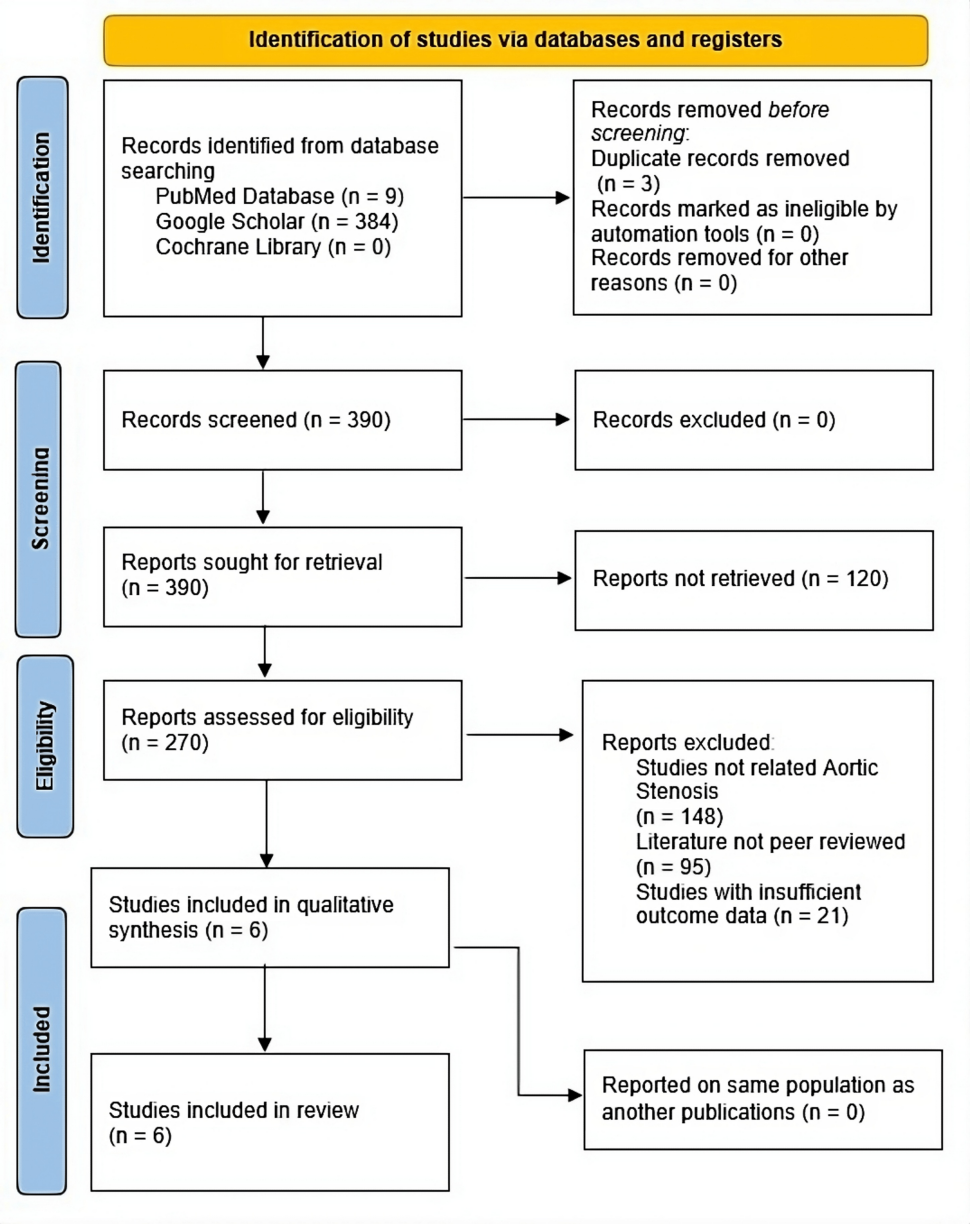
PRISMA flow chart

The PRISMA flow diagram in Figure 1 outlines the study selection process for the systematic review. Initially, 393 records were identified from three databases - PubMed (9), Google Scholar (384), and Cochrane Library (0). After removing three duplicates, 390 records were screened and sought for retrieval. During the retrieval phase, 120 reports could not be retrieved, leaving 270 reports for eligibility assessment. One hundred forty-eight studies were excluded as they used MRI imaging techniques for diagnosing other conditions unrelated to aortic stenosis, 95 for not being peer-reviewed, and 21 for insufficiency in data towards the proposed outcomes of this study. As a result, six studies were included in the qualitative synthesis. No further exclusions were made, and all six studies were ultimately included in the final review. The decision to include only non-randomized cohort studies stems from the lack of available RCTs specifically evaluating the role of 4DFM in valvular heart disease. Given that observational data from cohort studies can offer valuable insights into real-world patient outcomes, we prioritized these studies to capture a broader spectrum of clinical scenarios. A clear summary of sample characteristics, with demographics, gender distribution, the severity of AS, and clinical representativeness, along with inclusion and exclusion criteria of each study's sample, is represented in Appendix 1.

Quality Assessment

The quality assessment of the cohort studies shows variation in methodology and reporting rigor. Most studies clearly stated their research objectives and defined their study populations with sufficient participation rates. However, the majority lacked sample size justifications or power analyses, which weakened the reliability of the findings. Additionally, only a few studies examined different levels of exposure and assessed exposure more than once over time, increasing their methodological strength, such as Allen et al. [14]. The lack of sample size justification and power analysis in cohort studies affects the generalizability and interpretability of findings, potentially leading to inconclusive results and type II errors. Confounding variables such as age, sex, and comorbidities were often inadequately addressed, risking skewed results. Outcome measures were generally well-defined and reliable across studies, but there was limited information on blinding of outcome assessors, which could introduce measurement bias. Confounding variables were typically not adjusted for in most studies. The overall assessment of the studies shows an average score of eight, which indicates a "fair" quality being acceptable for the meta-analysis. While most studies scored poorly on sample size justification, they generally performed well on participant retention, indicating the need for a balanced approach to study design to enhance overall quality and reliability. The assessment is described in Appendix 2.

Risk Of Bias

**Figure 2:**
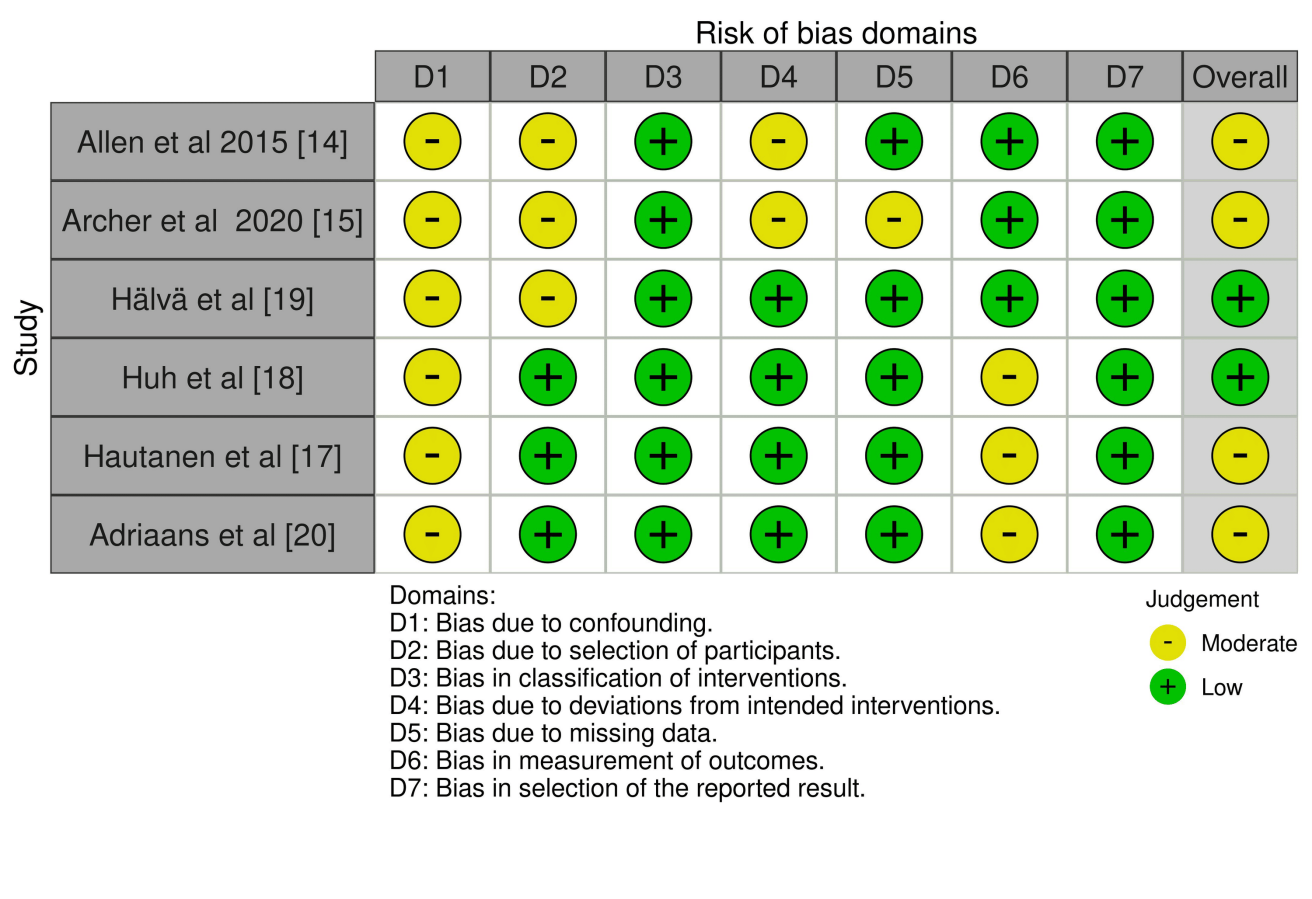
: Risk of Bias traffic plot for the studies included in the analysis

The Risk of Bias (ROB) traffic light plot in Figure 2 evaluates six studies across seven key domains, indicating the level of bias in each, providing a clear visual summary of the methodological robustness of the included studies. The domains assessed include confounding, selection of participants, classification of interventions, deviations from intended interventions, missing data, measurement of outcomes, and selection of reported results. Allen et al. [14] (2015) showed high bias in confounding (D1) and moderate bias in the selection of participants (D2) and missing data (D5), while Huh et al. [18] (2022) had a low risk across all domains. Archer et al. [15] (2020) and Adriaans et al. [20] (2020) exhibited higher risks in participant selection. Overall, most studies were categorized as having a low to moderate risk of bias, with no severe bias in most domains.

**Figure 3:**
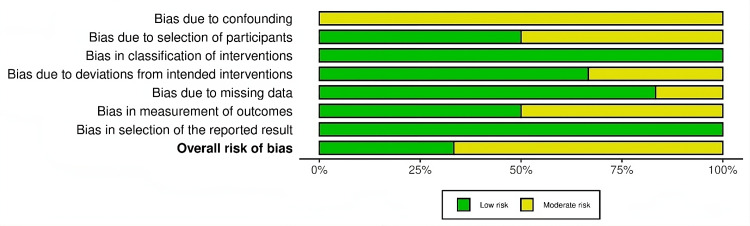
Overall risk of bias of the included studies

The bar graph in Figure 3 presents an overview of the ROB across several studies, evaluating seven key domains. The graph shows that bias due to confounding and participant selection predominantly carries moderate risk, while other domains, like classification of interventions and deviations from intended interventions, exhibit mostly low risk across the studies. For missing data, there is a balanced split between low and moderate risk. Bias in the measurement of outcomes and selection of reported results also lean heavily toward low risk, with a smaller portion classified as moderate. Overall, the majority of studies have a low risk of bias, particularly in outcome-related domains, although some moderate risk persists in participant selection and confounding.

The ROB indicates that certain studies, such as Allen et al. [14] and Hälvä et al. [19], exhibited a higher risk of bias in confounding (D1) and participant selection (D2) domains. These risks likely stem from inadequate adjustment for potential confounders or incomplete participant selection criteria, which can limit the comparability of outcomes across studies. Moderate risk depicts that some domains had incomplete reporting on key methods such as participant selection or randomization, which introduces bias. This collective risk profile reflects generally sound methodology but indicates minimal limitations in reliability and generalizability.

Mean Peak Flow Velocity

**Figure 4:**
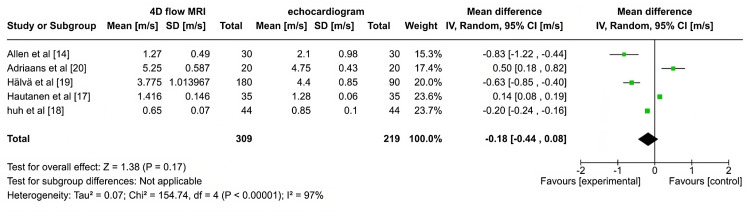
Forest plot for mean peak flow velocity

Figure 4 indicates the forest plots of the mean peak flow velocities measured by 4DFM and conventional methods. The observed value indicates the estimated 4DFM values are consistent with the compared methods. Allen et al. [14] and Hälvä et al. [19] study's negativity is likely due to the differences in imaging resolution or flow dynamics specific to their population and measurement settings. Similarly, Adriaans et al. [20] and Hautanen et al. [17] present positive mean differences, where 4DFM shows slightly higher peak velocities than echocardiography. This reflects methodological differences, such as specific adjustments in MRI processing and population characteristics that favor higher flow velocity measurements with 4DFM. The pooled mean difference of -0.18 m/s is likely minimal in clinical settings, supporting the use of either modality based on availability, patient desire, or clinical situations. The high I² value of 97% suggests substantial heterogeneity across the studies, indicating that the observed differences may stem from variations in study populations, measurement techniques, or specific settings rather than a consistent discrepancy between the two methods. Potential sources of heterogeneity include different patient demographics, variability in cardiac pathologies studied, and differing technical protocols for image acquisition and analysis.

Mean Pressure Gradient

**Figure 5:**
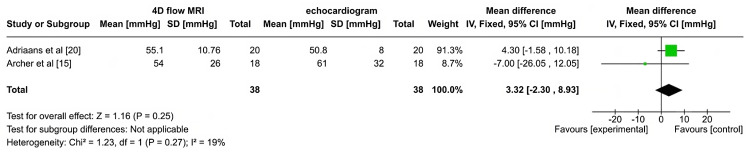
Forrest plot for mean pressure gradient

The forest plot in Figure 5 presents a comparison of the mean pressure gradient measurements (in mmHg) obtained using 4DFM and echocardiography for assessing valvular heart disease, specifically from two studies, Adriaans et al. [20] and Archer et al. [15]. Adriaans et al. [20] indicated the mean pressure gradient for 4DFM as 55.1 mmHg (SD=10.76) compared to TTE 50.8 mmHg (SD=8) for echocardiography. The mean difference between the modalities was 4.30 mmHg (CI: -1.58, 10.18). Archer et al. [15] reported the mean pressure gradient for 4DFM as 54 mmHg (SD=26), while for TTE as 61 mmHg (SD=32) with a mean difference of -7.00 mmHg (CI: -26.05, 12.05). The pooled result, combining both studies, shows a mean difference of 3.32 mmHg (CI: -2.30, 8.93), suggesting that 4DFM may measure pressure gradients slightly higher than echocardiography, though this difference is not statistically significant (p=0.25). Clinically, this implies that both approaches might be applied to accurately measure pressure gradients in valvular heart disease without having a major impact on treatment choices or patient care. Given the small variation in measurement results between the two modalities, practical considerations like patient preference, cost, or availability may influence the technique selection. The heterogeneity observed is low (I²=19%), indicating consistency between the studies. The results suggest no substantial advantage of either method for pressure gradient measurement in this context.

Aortic Valve Area

**Figure 6:**
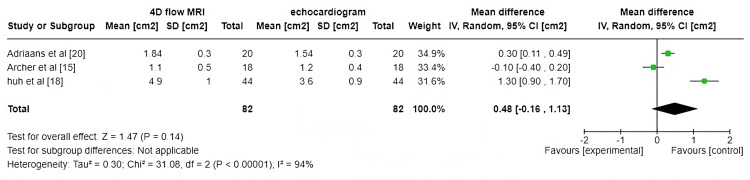
Forest plot for aortic valve area

This forest plot in Figure 6 compares measurements of the aortic valve area from two imaging modalities: 4DFM and echocardiogram. The three studies included in the meta-analysis are Adriaans et al. [20], Archer et al. [15], and Huh et al. [18], with respective sample sizes of 20, 18, and 42. Positive mean differences favor 4DFM, while negative values favor echocardiogram. Adriaans et al. [20] show a slight positive mean difference (0.30 cm² (0.11, 0.49)), favoring MRI. Archer et al. [15] have a small negative difference (-0.10 cm² (-0.40, 0.20)), favoring echocardiogram. Huh et al. [18] report a large positive difference (1.30 cm² (0.89, 1.71)), significantly favoring MRI. The pooled mean difference of 0.48 cm² (95% CI: -0.16, 1.12), though not statistically significant (p=0.14), may still hold clinical importance, particularly in borderline cases of aortic stenosis where precise measurement is critical. A larger measured valve area could influence treatment decisions, as one modality's greater sensitivity in detecting valve openings may provide a more accurate assessment of disease severity. This emphasizes the need for consistency in imaging protocols and measurement techniques to ensure reliable comparisons between 4D flow MRI and echocardiography in clinical practice. However, high heterogeneity (I²=93%) suggests substantial variation between studies, which could stem from differences in imaging protocols, such as variations in 4D flow MRI settings and echocardiographic techniques. Variability in sample characteristics, such as patient demographics, severity of valve disease, and differences in measurement protocols, like operator experience, contribute to this heterogeneity.

Inter-Observer Variability

**Figure 7:**
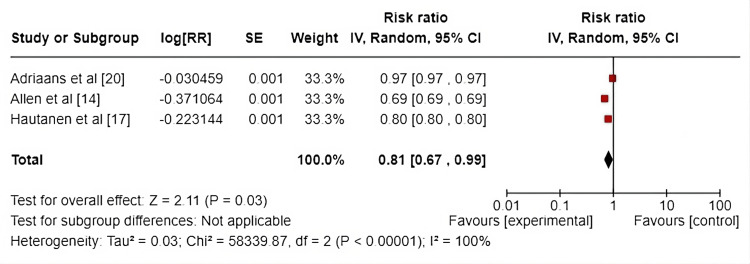
Forrest plot for interobserver variation

This forest plot in Figure 7 presents the results of a meta-analysis evaluating the risk ratio (RR) from three studies: Adriaans et al. [20], Allen et al. [14], and Hautanen et al. [17]. Each study's log-transformed risk ratio (log(RR)), standard error (SE), weight (33.3% each), and corresponding risk ratio (IV, random, 95% CI) are displayed. The use of log(RR) for measuring inter-observer variability across studies standardizes the comparative analysis and facilitates a clearer interpretation of differences in agreement. Log transformation helps manage data skewness, ensuring a more symmetric distribution for meta-analytic purposes. This approach allows the comparison of the likelihood of consistent assessments between intervention and control groups, offering a straightforward measure of reliability improvement across different study settings. The studies compare an experimental intervention against a control, and the risk ratio quantifies the likelihood of an event occurring in the experimental group relative to the control. Adriaans et al. [20] reported a risk ratio of 0.97 (95% CI: 0.97 to 0.97), suggesting near equivalence between the two groups. Allen et al. [14] found a risk ratio of 0.69 (95% CI: 0.69 to 0.69), indicating a 31% reduction in risk with the experimental intervention. Hautanen et al. [17] reported a risk ratio of 0.80 (95% CI: 0.80 to 0.80), suggesting a 20% risk reduction. The overall pooled risk ratio is 0.81 (95% CI: 0.67 to 0.99), with a statistically significant effect favoring the experimental group (p=0.03). The observed effect size suggests that the intervention could be associated with a meaningful reduction in the incidence of a specific event and improved assessment outcomes. However, the heterogeneity is extremely high (I²=100%), indicating considerable variability among the studies, which is due to differences in study design, sample population, and assessment protocols. This variability impacts the generalizability and clinical application of the findings. The black diamond at the bottom of the forest plot visually summarizes the overall effect size (0.81) and the 95% confidence interval (0.67 to 0.99). The position of the diamond's center represents the pooled risk ratio, while its width denotes the confidence interval range. The diamond lying to the left of 1 indicates that the results favor the experimental group over the control.

Discussion

Accuracy in measurement and assessment of the hemodynamic severity of AS is essential for making informed clinical decisions [27]. Various indices, including transvalvular peak velocities, pressure gradients, and AVA, have been proposed to evaluate the severity of AS [28,29]. The comparison of 4DFM with conventional imaging methods like TTE and 2D MRI for assessing AS highlights both its advantages and challenges. The observed mean differences in peak velocities and AVA highlight the nuanced diagnostic implications in clinical practice. Higher AVA values measured by 4DFM suggest that this method may better capture complex hemodynamic patterns without relying on geometric assumptions, improving diagnostic accuracy for specific cases, such as low-flow, low-gradient AS. Studies by Adriaans et al. [20], Archer et al. [15], and Huh et al. [18] showed that 4DFM often provides higher measurements for AVA and mean pressure gradients, offering superior spatial resolution and comprehensive flow analysis. However, findings varied, with Allen et al. [14] and Hälvä et al. [19] reporting underestimation of peak velocities compared to echocardiography. The high variability (I² >75%) reflects heterogeneity across many studies, likely driven by differences in patient demographics, AS severity, imaging planes, and software used for velocity calculations. These variations underscore the challenge of generalizing findings to broader populations. Factors like patient anatomy, technical expertise, and equipment quality also contribute to inconsistency.

The comparison of peak flow velocities between 4DFM and conventional imaging methods (such as echocardiography and 2D MRI) highlights the nuances in diagnostic accuracy for AS. The meta-analysis demonstrates that, while echocardiography often reports higher peak velocities compared to 4DFM (mean difference of -0.18 m/s), the variation between studies is significant (I² = 97%). For instance, Allen et al. [14] and Hälvä et al. [19] report substantial underestimation by 4DFM, whereas Adriaans et al. [20] find the opposite, showing that 4DFM can sometimes overestimate peak velocities. In contrast, studies like Hautanen et al. [17] and Huh et al. [18] report smaller discrepancies, with good agreement between 4DFM and 2D techniques in certain anatomical locations like the aortic root or tubular aorta. These variations can be attributed to differences in patient populations, imaging planes, and software algorithms used for velocity calculations. Despite the heterogeneity, 4DFM remains valuable for providing detailed hemodynamic information. Its ability to measure complex flow patterns offers advantages over echocardiography in detecting subtle flow anomalies, although the latter remains more commonly used for measuring peak velocities. Ultimately, combining 4DFM with traditional methods could enhance diagnostic precision, offering complementary insights that may improve clinical decision-making in AS management.

In comparing the efficacy of 4DFM with echocardiography for assessing AS, both imaging techniques offer distinct advantages, though their relative effectiveness depends on the clinical context. In the studies by Adriaans et al. [20] and Archer et al. [15], 4DFM demonstrated the potential to provide more comprehensive, three-dimensional assessments of blood flow dynamics across the aortic valve, while echocardiography-specifically Doppler transthoracic echocardiography (TTE)-remains a widely used, non-invasive standard. The statistical findings from these studies revealed that 4DFM tends to measure slightly higher mean pressure gradients compared to echocardiography, as observed in Adriaans et al. [20] (mean difference: 4.30 mmHg, 95% CI: -1.58, 10.18) and Archer et al. [15] (mean difference: -7.00 mmHg, 95% CI: -26.05, 12.05). Despite this, the overall pooled results showed no significant difference between the two modalities (p=0.25), indicating that both methods are clinically reliable for assessing pressure gradients in AS. The main advantage of 4DFM is its ability to assess complex flow patterns and provide a detailed spatial understanding of aortic valve hemodynamics, which could be critical in more nuanced cases or for surgical planning. However, echocardiography, with its ease of use, cost-effectiveness, and accessibility, continues to be the frontline tool in clinical settings. For routine assessments, TTE is often sufficient, though in cases where a more detailed analysis of flow patterns is required or when echocardiographic results are inconclusive, 4DFM can offer additional insight. In terms of reproducibility and reliability, both techniques showed strong correlations with invasive catheter measurements of pressure gradients, though Doppler TTE tended to overestimate peak pressure gradients in comparison to invasive data (Archer et al. [15], p=0.0002). In contrast, 4DFM showed better agreement with invasive catheter evaluations, suggesting that it may be a more accurate method in certain cases. Additionally, 4DFM has demonstrated better consistency in assessing AVA, with larger AVA measurements compared to echocardiography, likely due to differences in left ventricular outflow tract (LVOT) area measurements.

The assessment of AVA using 4DFM has shown considerable promise in accurately diagnosing aortic stenosis (AS) compared to conventional methods like 2D phase-contrast MRI (PC-MRI) and Doppler transthoracic echocardiography (TTE). In studies like Adriaans et al. [20] and Archer et al. [15], 4DFM generally yields higher AVA values due to its superior spatial resolution and ability to account for complex flow dynamics without geometric assumptions. For instance, Adriaans et al. [20] reported a positive mean difference of +0.30 cm² in AVA favoring 4DFM over TTE, while Huh et al. [18] reported a significantly higher difference of +1.30 cm², suggesting 4DFM's greater efficacy in specific cases. However, the variability in these findings, as indicated by the high heterogeneity (I²=93%) in the meta-analysis, underscores the challenges in standardizing AVA measurements across different modalities. While Archer et al. [15] reported minimal difference between 4D flow and TTE (bias = -0.10 cm²), other studies demonstrate larger discrepancies, particularly in severe AS cases where 2D PC-MRI often underestimates peak flow velocity and pressure gradients. Despite these variations, 4DFM provides more detailed hemodynamic insights, making it a valuable tool for comprehensive AS assessment, particularly in challenging cases like low-flow, low-gradient AS, where geometric independence plays a critical role in diagnosis.

The studies by Adriaans et al. [20], Allen et al. [14], and Hautanen et al. [17] provide important insights into the reproducibility and reliability of 4DFM for assessing AS. Intra- and interobserver agreement are key factors in determining the diagnostic accuracy of any imaging modality. Adriaans et al. [20] demonstrated excellent interobserver agreement for peak velocity (Vpeak) with intraclass correlation coefficients (ICCs) of 1.00, which emphasizes the robustness of 4DFM in clinical assessments. Similarly, Hautanen et al. [17] reported high interobserver agreement for peak velocity (ICC=0.8) in the tubular aorta, though the agreement was notably lower in the aortic root, indicating a potential area for improvement.

In the context of the meta-analysis, which indicated high heterogeneity (I²=100%) but a pooled risk ratio favoring the experimental group (RR=0.81), the diagnostic accuracy of 4DFM in assessing AS appears promising. However, variability in measurements across studies suggests that while 4DFM excels in precision and interobserver reliability, it may be influenced by specific anatomical or technical factors that differ between studies. Comparatively, conventional modalities like 2D MRI and TTE, while showing good correlations with 4DFM, tend to either underestimate (2D MRI) or provide less spatial resolution (TTE), leading to lower accuracy in certain AS measurements, such as peak velocity and AVA. The meta-analysis forest plot highlights the variability across imaging techniques, with 4DFM generally outperforming TTE in terms of sensitivity to flow dynamics, though it requires further validation in larger, more standardized studies.

Overall, while echocardiography remains the gold standard for initial assessments, 4DFM presents a valuable complementary tool, particularly for complex or severe cases requiring detailed flow analysis. Its efficacy lies in its ability to provide a more comprehensive, 3D hemodynamic picture, though practical factors like cost and availability continue to limit its widespread use.

Despite these discrepancies, 4DFM demonstrated better agreement with invasive catheter measurements, particularly in complex AS cases. Interobserver reliability was excellent in several studies, but variability in results (high heterogeneity) suggests that anatomical and technical factors can affect diagnostic accuracy. Overall, while 4DFM enhances detailed hemodynamic assessment, it remains a valuable complementary tool to echocardiography, particularly for nuanced or inconclusive cases. Furthermore, The technique of 4D flow MRI offers the opportunity to derive advanced hemodynamic measures such as vorticity and helicity, wall shear stress, flow displacement, pressure gradients, viscous energy loss, and turbulent kinetic energy [16]. Although primarily used in larger academic centers, the recent reduction in scan times to under 10 minutes and the introduction of commercial 4DFM processing software have facilitated its clinical translation, allowing 4DFM to be incorporated into standard cardiothoracic MRI protocols with data analysis integrated into routine clinical workflows [12]. Clinically, 4DFM is particularly beneficial for patients with inconclusive echocardiographic results or complex AS cases, offering advanced insights like wall shear stress and turbulent kinetic energy. However, limitations such as cost, availability, and longer post-processing times must be acknowledged, emphasizing its role as a complementary, rather than primary, imaging modality.

The present study on the feasibility of 4DFM in quantifying aortic stenosis has a few limitations. First, the studied samples in the reviewed studies are quite small; many cohorts represent less than 100 participants. This reduces the statistical capability and sample generality of the results. Secondly, great variation is observed in terms of patient sampling and imaging methods. This variability may change the reproducibility of the outcome, such as peak velocity and aortic valve area. Third, the practical clinical application of 4DFM technology and its assessment is currently less generalized than that of echocardiography due to the limited access to this technology and specialists in specific clinical settings. Moreover, 4DFM is slightly more expensive and elaborate than other MRI readings, which are also time-consuming. Fourth, we acknowledge the potential limitation of not including the Embase database, which may exclude some studies, particularly those published in European journals. This selection approach aimed to balance resource availability with relevance to the study's focus on aortic stenosis imaging. Finally, pressure gradients are given as uncertain, and for that reason, it is concluded that there could currently be a clear need for standardization of protocols to provide more distinct results for various patient groups and specific clinical environments. Despite these disadvantages, the study demonstrates a well-rounded approach through a thorough analysis of key components, ensuring reliability and providing valuable technical insights. The robust assessment and detailed evaluation highlight the practical relevance, aligning with clinical needs and supporting applicability in real-world scenarios. This careful integration of comprehensive review and focused technical examination strengthens the depth and credibility of the work while maintaining clear, actionable conclusions for clinical practice.

Future research should focus on addressing the current gaps and limitations of 4DFM in aortic stenosis evaluation. Larger, multicenter studies with diverse populations are needed to validate the findings and enhance the generalizability of 4DFM as a diagnostic tool. Additionally, efforts should be made to standardize imaging protocols, particularly regarding velocity measurements and aortic valve area calculations, to reduce variability across studies. Investigating the cost-effectiveness of 4DFM and its impact on clinical decision-making, particularly in comparison to conventional methods like echocardiography, is also essential. Further exploration of advanced hemodynamic metrics such as vorticity, helicity, and turbulent kinetic energy may provide deeper insights into disease progression and prognosis. Integrating artificial intelligence and machine learning for image analysis could also streamline the interpretation of 4DFM data, making it more accessible for routine clinical use.

## Conclusions

The present study underscores the growing potential of 4DFM as an advanced imaging tool for AS assessment. While not yet a universal standard, 4DFM shows promise due to its ability to provide high spatial resolution, superior visualization of flow patterns, and reliable AVA measurements. Compared to conventional methods like TTE and 2D MRI, 4DFM captures complex hemodynamics more effectively, benefiting surgical planning and management in complex AS cases. Despite its advantages, challenges such as high costs and the need for specialized training limit widespread adoption. Advances in technology, including shorter scan times and user-friendly software, are gradually enhancing its clinical feasibility. Based on the findings, while 4DFM is not yet fully applicable in most clinics, they point to the fact that it could potentially change diagnostic approaches, especially in cases in which regular techniques prove insufficient. Since its implementation, 4DFM is expected to elevate its position in the process of stratification of valvular heart diseases, thus contributing in the improvement of patient outcomes besides being useful in future standard operating procedures. Integrating 4DFM with established techniques could lead to improved diagnostic accuracy and patient stratification, supporting more informed decision-making. Future work should focus on larger, multi-center studies and standardizing imaging protocols to solidify 4DFM's role in clinical practice, ensuring it complements TTE, particularly when conventional results are inconclusive.

## Appendices

**Table 3:** Appendix 1 - sample characteristics of the included studies NA - not available; AS - aortic stenosis; SD - standard deviation; M - male; F - female; SAVR - surgical aortic valve replacement; TAVI - transcatheter aortic valve implantation; MRI - magnetic resonance imaging; CABG - coronary artery by-pass grafting; BAV - bicuspid aortic valve; TAV - tricuspid aortic valves; 4DFM - 4D flow MRI; NYHA - New York Heart Association

Study	Country	Sample Size	Mean Age (± SD) in Years	Gender Distribution	AS Severity	Inclusion Criteria	Exclusion Criteria
Huh et al. [18]	United States of America	44 (37 controls, 7 AS)	Controls: 51.9 (± 18.2), AS: 64.2 (± 9.6)	20 F, 1 F (AS)	Moderate to Severe	Healthy volunteers and patients with moderate-to-severe AS. Tests performed on same day with caffeine restrictions.	None specified for controls; AS patients excluded with significant other valvular disease.
Halva et al. [19]	Finland	90 AS patients	73.3 (± 11.3)	51 M, 39 F	Severe	Patients referred for aortic valve replacement, either open surgery or TAVI.	Excluded with other significant valvular disease, incompatible implants, severe obesity, or inability to lie flat for 4DFM.
Archer et al. [15]	United Kingdom	18 AS patients	TAVI:82(NA), SAVR:68(NA)	4 M, 14 F	Severe AS	Patients with severe AS confirmed on echocardiography, undergoing pre-intervention 4D flow MRI and follow-up post-intervention.	Excluded with moderate/severe aortic regurgitation, significant other valve disease, coronary artery disease needing CABG, MRI contraindications, or inability to perform 6 minute walk test.
Allen et al. [14]	United States of America	30 BAV patients	13.9 ( ± 4.4)	17 M, 13 F	Moderate	Pediatric and young adult patients with BAV and various indications for cardiovascular MRI.	Patients unable to cooperate or requiring general anesthesia. Z-scores calculated for age and size normalization.
Adriaans et al. [20]	Netherlands	20 AS patients	69.3 (± 5.0)	13 M, 7 F	Severe	Adult AS patients attending clinic for echocardiographic follow-up.	Excluded with prior aortic valve replacement, NYHA class ≥3 heart failure, renal failure, metallic implants, claustrophobia, or atrial fibrillation.
Hautanen et al. [17]	Finland	83 AS patients (35 BAV, 48 TAV)	53.7 (± 15.3)	58 M, 25 F	Severe	Patients with BAV or TAV, having MRI scans in Kuopio University Hospital, including 4D flow MRI.	Excluded with prosthetic aortic valve, aortic root replacement, poor image quality, or MRI contraindications.

**Table 4:** Quality assessment of the cohort studies Y - yes; N - no; ? - not estimated/ unknown

Criteria	Hautanen et al. (2023) [17]	Adriaans et al. (2020) [20]	Archer et al. [15]	Allen et al. [14]	Hälvä et al. [19]	Huh et al. [18] (2021)
1. "Was the research question or objective in this paper clearly stated?"	Y	Y	Y	Y	Y	Y
2. "Was the study population clearly specified and defined?"	Y	Y	Y	Y	Y	Y
3. “Was the participation rate of eligible persons at least 50%?"	Y	Y	?	Y	Y	Y
4. "Were all subjects selected or recruited from the same or similar populations? Were inclusion/exclusion criteria prespecified and applied uniformly?"	Y	Y	Y	Y	Y	Y
5. "Was a sample size justification, power description, or variance and effect estimates provided?"	N	N	N	N	N	N
6. "Were exposures measured prior to outcomes?"	Y	Y	Y	Y	Y	Y
7. "Was the timeframe sufficient to reasonably expect an association?"	Y	Y	Y	Y	Y	Y
8. "Did the study examine different levels of exposure (categories or continuous variables)?"	N	N	N	Y	N	N
9. "Were exposure measures clearly defined, valid, reliable, and consistently applied?"	Y	Y	Y	Y	Y	Y
10. "Was the exposure assessed more than once over time?"	N	N	N	Y	N	N
11. "Were outcome measures clearly defined, valid, reliable, and consistently applied?"	Y	Y	Y	Y	Y	Y
12. "Were outcome assessors blinded to the exposure status of participants?"	N	Y	?	?	?	Y
13. "Was loss to follow-up after baseline 20% or less?"	Y	Y	Y	Y	Y	Y
14. "Were key potential confounding variables measured and adjusted for statistically?"	N	N	N	Y	N	N
Total Score	9	10	8	12	9	10
Overall Quality	Fair	Fair	Fair	Good	Fair	Fair
